# On the Perforation of the Intestinal Canal by Worms

**Published:** 1851-10

**Authors:** 


*On the Perforation of the Intestinal Canal by Worms.* By Dr. Buchner.—The perforation of the intestinal canal by lumbricoids has often been denied. In No. 1 of the 'Med. Zeitung,' for 1850, however, Dr. Köppe relates a case in which such perforation seems to have resulted; and in the present note Dr. Buchner relates another. A strongly made woman, somewhat advanced in years, died in from eight to ten hours after the appearance of symptoms of acute peritonitis. On examination, the intestinal contents were found effused, and a large *ascaris lumbricoides* was lying in the immediate vicinity of the duodenum. In this gut there was observed an exactly circular hole, having sharply cut borders, and presenting an appearance unlike any ever seen in the intestinal canal by the author. It was accompanied neither internally nor externally by any signs of inflammation or other diseased appearance. No other cause of perforation could be discovered; and within the duodenum other lumbricoids were found.—*Ibid, from Med. Zeitung,* No. 28.

